# Supplementary material for: Nonlinear co-generation of graphene plasmons for optoelectronic logic operations
Source: Nat Commun. 2022 Jun 6;13:3138. doi: 10.1038/s41467-022-30901-8 (PMC9170737; doi:10.1038/s41467-022-30901-8)
Supplement: Supplementary file 1 — Supplementary Information [file 41467_2022_30901_MOESM1_ESM.docx]

*Supplementary Information for*

**Nonlinear co-generation of graphene plasmons for optoelectronic logic operations**

Yiwei Li^1,#^, Ning An^1,#^, Zheyi Lu^2,#^, Yuchen Wang^1^, Bing Chang^1^, Teng Tan^1,3^, Xuhan Guo^4^, Xizhen Xu^5^, Jun He^5^, Handing Xia^6^, Zhaohui Wu^6^, Yikai Su^4^, Yuan Liu^2,^*, Yunjiang Rao^1,3,^*, Giancarlo Soavi^7,8^*, Baicheng Yao^1,^*

*^1^Key Laboratory of Optical Fibre Sensing and Communications (Education Ministry of China), University of Electronic Science and Technology of China, Chengdu, China.*

*^2^Key Laboratory for Micro-Nano Optoelectronic Devices (Education Ministry of China), School of Physics and Electronics, Hunan University, Changsha, China.*

*^3^Research Centre for Optical Fibre Sensing, Zhejiang Laboratory, Hangzhou, China.*

*^4^State Key Laboratory of Advanced Optical Communication Systems and Networks, Shanghai Jiao Tong University, Shanghai, China.*

*^5^Guangdong and Hong Kong Joint Research Center for Optical Fiber Sensors, Shenzhen University, Shenzhen, China.*

*^6^Research Center of Laser Fusion, China Academic of Engineering Physics, Mianyang 621900, China.*

*^7^Institute of Solid State Physics, Friedrich Schiller University Jena, Jena, Germany.*

*^8^Abbe Center of Photonics, Friedrich Schiller University Jena, Jena, Germany.*

*^#^These authors contributed equally.*

**Corresponding authors:*

[*yaobaicheng@uestc.edu.cn*](mailto:yaobaicheng@uestc.edu.cn)*;* [*giancarlo.soavi@uni-jena.de*](mailto:giancarlo.soavi@uni-jena.de)*;*

[*yuanliuhnu@hnu.edu.cn*](mailto:yuanliuhnu@hnu.edu.cn)*;* [*yjrao@uestc.edu.cn*](mailto:yjrao@uestc.edu.cn)

**Supplementary Note 1. Theoretical analysis.**

Both the pump and probe pulses propagate inside the fiber and interact with graphene via evanescent field. The DFG relies on the in-fiber phase matching, hence we first investigate the effective refractive index of the GDF geometry for the optical mode (TM polarized HE_11_). Supplementary Figure 1a shows the modelling of the cross-sectional view of our GDF. The material of the fiber is silica, with refractive index of the core/cladding of 1.452/1.446. On the D-plane and close to the core, the 0.5 nm thick graphene is modelled as an ultrathin metal, with intraband and interband conductivities [1,2,3]

$\sigma_{g,intra}=\frac{ie^{2}E_{F}}{\pi\hbar(2\pi f+\frac{i}{\tau})}$ (S1-A)

$\sigma_{g,inter}=\frac{ie^{2}E_{F}}{4\pi\hbar}ln\left[ \frac{2\left| E_{F} \right|-\hbar(2\pi f+\frac{i}{\tau})}{2\left| E_{F} \right|+\hbar(2\pi f+\frac{i}{\tau})} \right]$ (S1-B)

Here *E_F_* is the Fermi level, *τ* = 10^-13^ s is the scattering time, *T* is the temperature, *f* is the frequency, *ħ* = 1.05×*10^-34^* eV·s is the reduced Planck constant, *k_B_* = 1.38×*10^-23^J*/K is the Boltzmann’s constant, and *e = -1.61×10^-19^C* is the unit charge. Such conductivity is a complex number, and its effective permittivity and refractive index are determined by its complex conductivity:

$\left\{ \begin{aligned} \epsilon_{g}=\frac{-\sigma_{g,i}+i\sigma_{g,r}}{2\pi f\Delta} \\ \left( n_{g,r}+in_{g,i} \right)^{2}=\epsilon_{g,r}+i\epsilon_{g,i} \end{aligned} \right.$ (S2)

For propagating optical modes, *n_g,r_* influences the phase velocity while *n_g,i_* refers to the propagation loss. For example, for light with wavelength of 1600 nm and a Fermi level of 0.2eV, |*n_g_*| is ≈ 3.61. The calculation of the graphene’s dispersion was discussed in details in previous studies [4].

By using the finite-element method with the commercial software COMSOL multiphysics, we simulated the electrical field distributions of the GDF (core diameter 6 μm) for several optical wavelengths (Supplementary Figure 1b maps). In Supplementary Figure 1c, we plot the calculated frequency dependent effective refractive index (or the dispersion) of the TM-HE_11_ mode in the GDF. A lower effective refractive index enables a higher overlap between light and graphene. A higher optical frequency also corresponds to a higher effective refractive index (and thus lower overlap): in particular for 1550 nm wavelength (192 THz) we have 3% optical energy overlaps with graphene, while for 1923 nm wavelength (156 THz) we have 8% optical energy overlaps with graphene.

Supplementary Figure 1 | Optical transmission in the GDF. a, Schematic geometry of the GDF. b, Simulated electrical field distributions of the fundamental mode (TM polarized HE_11_) in the GDF. c, Correlation of the optical frequency and the effective refractive index of the D-shaped fiber.

Then we discuss the χ^(2)^ nonlinearity of graphene on the D-shaped fiber, and the phase matching condition to be satisfied for the plasmonic generation. For the DFG based plasmon generation, the pump photons (*f_pump_* in the C band) are converted into probe photons (*f_probe_* in the C band) and plasmons (*f_sp_* in the THz band). During this process, also the optical momentum must be conserved (phase-matching). Considering *k = 2π/λ = 2πn_eff_/cT = 2πfn_eff_/c*, where *n_eff_* is the effective refractive index and *c* is the speed of light in vacuum, with the counter-propagating pump-probe geometry, we write the energy conservation and phase-matching conditions as

$\left\{ \begin{matrix} f_{probe}n_{probe}-f_{sp}n_{sp}=-f_{pump}n_{pump} \\ f_{probe}+f_{sp}=f_{pump} \end{matrix} \right.$ (S3)

Here *n_p_*, *n_s_*, and *n_sp_* are the effective indexes of the pump, probe and the plasmon respectively. To satisfy the phase-matching condition, *f_pump_*, *f_probe_* and *n_sp_* should be selected and adjusted carefully to achieve:

$(n_{SP}+n_{probe})f_{probe}=f_{pump}(n_{SP}-n_{pump}),n_{SP}\gg n_{p}$ (S4)

Besides the phase-matching, the resonant dispersion of graphene also plays a key role in the plasmonic generation: graphene’s plasmons can only be excited in the polariton resonances, where *k_sp_* and *f_sp_* follow the Euler equation of motion. For ordinary Schrödinger fermions with mass *m*_b_, the Drude weight is given by *D* = π*e*^2^*n*/*m*_b_. Without considering any phonon coupling, the Drude weight of (non-interacting) massless Dirac fermions is given by *D* = 4*E_F_σ_uni_*/*ħ* [5]. Hence one can write the Drude curve as

$f_{sp}=\frac{1}{2\pi}\sqrt{\frac{8E_{F}\sigma_{uni}k_{SP}}{\mathcal{\hbar E}}}$ (S5)

Here *ε* is the conductivity of the medium. Another important condition to realize the co-excitation of multiple plasmon-polaritons with different frequencies is the plasmon-phonon coupling, as a plasmon-phonon interaction in the dispersion map splits the Drude curve into two branches [6,7]. For graphene deposited on silica, one should consider two phonons: the longitudinal optical phonon with *f_LO_* = 24 THz, and the surface optical phonon with *f_SO_* = 36 THz [8]. Supplementary Figure 2a-2c plot the ‘*f_sp_-k*’ dispersions when |*E_F_*| = 0.1 to 0.3 eV.

Considering the linear response theory and within the random phase approximation, one can calculate the imaginary part of the Lindhard function, which determines the lifetime of the plasmons. By using the simplified random phase approximation method [9], one can write the polariton permittivity of graphene *ε_RPA_(f_sp_,***k***) = (ε_1_+ε_2_)/2-2πe^2^/***k***П(f_sp_,***k***)*, here *ε_1_* and *ε_2_* are upper and lower media permittivity (in our case, air and silica fiber), and *П(f_sp_,***k***)* is the polarizability. For finite doping, *П(f,***k***)* could be written as

$\Pi(f_{sp},\mathbf{k})=\frac{2|E_{F}|}{\pi v_{F}}\left[ 1-\frac{2\pi f_{sp}}{\sqrt{{(2\pi f_{sp})}^{2}-{v_{F}}^{2}k^{2}}} \right]$ (S6)

Supplementary Figure 2 | Plasmon dispersion and phase matching of GDF. a-c, Calculated frequency-momentum dispersions for graphene plasmons. Blue curves show the Drude dispersion of graphene, the red lines show the DFG phase matching condition when *f_pump_* = 192 THz. The longitudinal optical (LO) and surface optical (SO) phonon modes are marked as dashed lines at frequencies 24 THz and 36 THz. d-f, Simulated RPA maps of graphene for Fermi levels. The color bar is the normalized 1/|*Im[ε_RPA_(f_sp_,***k***)]|*.

The imaginary part of *ε_RPA_(f_sp_,***k***)* describes the resonant intensity of the plasmons. Supplementary Figure 2d-f map the 1/|*Im[ε_RPA_(f_sp_,***k***)]|* for different doping values. In the RPA analysis, the influence of electron-phonon interactions on the polariton permittivity *ε_RPA_(f_sp_,***k***)* is included as

$\varepsilon_{RPA}\left( f_{sp},\mathbf{k} \right)=1-\frac{2\pi e^{2}}{\varepsilon_{1}\boldsymbol{k}}\Pi\left( f_{sp},\mathbf{k} \right)+\frac{\alpha e^{-2kd}}{1-\alpha e^{-2kd}-\left( 2\pi f_{sp} \right)^{2}/{(2\pi f_{ph})}^{2}}$ (S7)

Here *d* is the distance between the graphene layer and the substrate while *α* ≈ 1/6 is the air-silica dielectric mismatch.

In addition, also the second order nonlinear susceptibility *χ^(2)^* of graphene on a silica substrate plays a key role for plasmonic generation *via* DFG. As discussed in Ref. [10], for light propagating with a wavevector **k** parallel to the graphene plane and *f_pump_* ≈ *f_probe_*, the effective *χ^(2)^* [11], determined by the geometrical asymmetry, can be written as

${\chi_{eff}}^{\left( 2 \right)}=\frac{e^{3}}{{8\pi}^{2}\hbar^{2}}\frac{1}{kf_{pump}f_{sp}}[\frac{\pi}{2}+\arctan(\frac{2\pi f_{pump}-2v_{F}\sqrt{2m_{e}E_{F}}}{\gamma})]$ (S8)

Here *ħk_F_* = *ħ(2m_e_E_F_)^1/2^* *k_F_* is the Fermi momentum, and *γ* is the scattering ratio. We map the simulated *χ_eff_^(2)^* in Supplementary Figure 3a, with a fixed *f_pump_* = 192 THz. We note that a higher *E_F_* leads to a lower *χ_eff_ ^(2)^*, but a higher *E_F_* enables a smaller 1/|*Im[ε_RPA_(f_sp_,***k***)]|*.

Beside the nonlinear gain, we also show the plasmon coupling based loss *L_SP_(f_sp_,***k***)* (corresponding to the *Im[ε_RPA_(f_sp_,***k***)]*) along the GDF, with consideration of the phonon couplings. Typically, the *L_SP_(f_sp_,***k***)* is determined by both the carrier mobility and the Fermi level of graphene [12].

$L_{SP}\left( \mathbf{k},f_{sp} \right)=-Im\left\{ 1-\frac{e^{2}}{2\boldsymbol{k}\epsilon_{1}}\psi-\sum_{j} f_{ph,j}\psi\right\}$ (S9)

$\psi=-\frac{g_{s}}{4\pi^{2}}\sum\int\frac{f_{d}\left( \epsilon_{s} \right)-f_{d}\left( \epsilon_{s\boldsymbol{k}} \right)}{2\pi f_{sp}\hbar+\frac{i\hbar}{\tau}+\epsilon_{s}-\epsilon_{s\boldsymbol{k}}}d\mathbf{k}F(s,\mathbf{k})$ (S10)

Here *f_ph,j_* is the phonon resonances, *g_s_ =* 4, *f_d_(ϵ)* the Fermi-Dirac distribution, *ϵ_s_ = sv_F_*, *ϵ_s_***_k_** *= sv_F_***k**, *s* = ±1, *F(s,***k***)* is the band overlap function of Dirac spectrum, which equals 1 for the waveguide geometry. In the simplified case without considering the phonon effects, we plot the phase matched ‘|*E_F_*|-*L_sp_*’ correlation for the fixed pumping wavelength 1560 nm, as Supplementary Figure 3b shows.

__

Supplementary Figure 3 | Parameters related to the nonlinear generation of graphene plasmons. a, 2^nd^-order nonlinear susceptibility of the GDF. Here we map the *χ_eff_^(2)^* for different values of *f_sp_* from 5 to 45 THz, increasing *E_F_* from 0.1 to 0.3 eV and a fixed pump frequency of 192 THz. In the color bar, *χ_eff_^(2)^* changes from 5.6×10^-7^ esu to 7.1×10^-7^ esu. b, Under phase matching condition, *L_sp_* decreases with the increment of |*E_F_*|.

**Supplementary Note 2. Nanofabrication and characterization of the device.**

Supplementary Figure 4 shows the nanofabrication steps for the GDF device. In *step 1*, a commercial single mode silica fiber (Corning RC-1550) is utilized, with fiber core diameter 6 μm and transmission loss < 0.5 dB/km at 1550 nm. For better nanofabrication and to increase its stability, the fiber is fixed on a glass substrate. The fiber is then mechanically side-polished in *step 2*, under an optical microscope and an on-line transmission power monitor. After polishing, we obtain a D-shaped fiber. The length of the D-shaped region is 2 mm and the surface of the D-shaped region is smooth on a nm scale. This guarantees that the scattering losses induced by the process are negligible [13]. In the D-shaped fiber, the distance between the core and the air is almost 0, ensuring strong light-graphene interaction. In *step 3* and *step 4*, a chemical vapor deposition (CVD) grown graphene monolayer is transferred onto the chip using PMMA and the wet-transfer method [14]. The PMMA is then removed using acetone. Next, in *step 5* and *step 6*, the Ti/Au (20/50 nm) contact pads (source and drain) are deposited on the D-shaped fiber by electron beam evaporation. When tuning the voltage between these two gold contacts, the Fermi level of graphene can be modulated. Supplementary Figure 4b shows the optical microscope images of the device.

Supplementary Figure 4 | Device fabrication. a, Fabrication steps. b, Top-view optical microscope images, from left to right: silica D-shaped fiber; the GDF transmitting red light (633 nm); GDF with electrodes, the width of the Au-graphene-Au transistor channel is 200 μm.

In Supplementary Figure 5a, we plot the measured optical transmission loss of our GDF device in the range 1500 nm to 1600 nm, obtained using a continuous-wave tunable laser (Santec TSL-710). In this measurement, we fix the average launched-in optical power to 1 mW (far below the Pauli blocking threshold to avoid saturable absorption) and vary the driving voltage. In the spectrum, the higher loss at lower frequencies (longer wavelengths) is due to the stronger evanescent scattering. Besides, when changing the driving voltage on the GDF, light transmission can be considerably tuned. This is determined by the electrically tunable nonlinear absorption. Such a phenomenon has been widely used in graphene based optoelectronic modulators [15]. In Supplementary Figure 5b, we plot the correlation between the driving voltage and the measured transmission for a fixed laser wavelength of 1560 nm. The transmission of our GDF decreases first from 26.7% (0 V, *E*_F_ ≈ 0.1 eV) to 23.2% (0.25 V, *E*_F_ ≈ 0 eV), and finally increases back to 88.1% (> 1.25 V, *E*_F_ > 0.4 eV). This process is due to the interband photoelectron transition. At least 10% (≈ 0.5 dB) of the losses is induced by the linear absorption and scattering of the D shaped fibre.


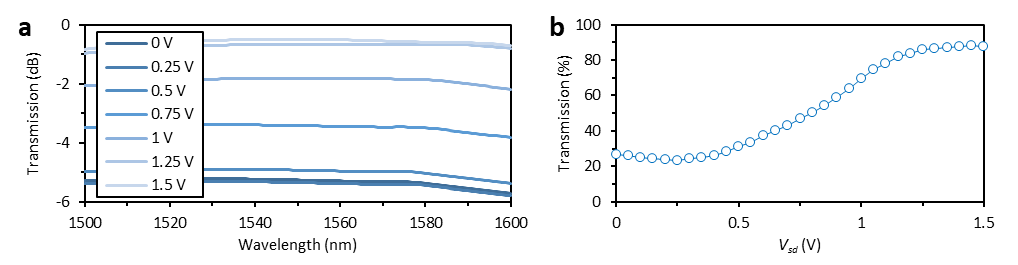


Supplementary Figure 5 | Optical transmission measurement. a, Measured transmission spectrum of the GDF in the range 1500 nm to 1600 nm. b, Electrically tunable transmission in the GDF at 1560 nm.

We now discuss the electrical properties of the GDF device (Supplementary Figure 6). The possibility of tuning the Fermi level in a two-terminal device is a unique feature of graphene: its ambipolar behaviour results in a “kink” in the *I-V* curve [16]. Due to the external voltage (electron injection), the p-doped graphene on the fiber (SiO_2_ substrate) becomes first neutral and subsequently n-doped. Thus, the “kink” corresponds to the transition from n-transport to p-transport across the Dirac point, suggesting that the resistance (or the carrier density) can be tuned by changing the source-drain voltage [17,18]. The blue curve in Supplementary Figure 6a plots the I-V curve of our device. This is clearly nonlinear: when the driving voltage *V_sd_* is negative (*V_sd_ << V_kink_*), the current is carried by holes throughout the length of the channel (Region I). When *V_sd_ ≈ V_kink_*, the vanishing carrier density produces a ‘pinch-off’ region at the drain (region II) that renders the current in the channel relatively insensitive to *V_sd_* and results in the pronounced kink seen in the I–V characteristic. Here the graphene is close to the Dirac point. For *V_sd_ >> V_kink_*, the minimal density point resides in the channel, producing a pinch-off region that moves from source to drain with increasing voltage (region III). In this bias range the carriers in the channel on the source side of the minimum density point are holes, and those on the drain side are electrons. In this ambipolar regime, the pinch-off point becomes a place of recombination for holes flowing from the source and electrons flowing from the drain. In the bottom panel of Supplementary Figure 6a, we show the zoomed-in curve when *V_sd_* is in a small range -0.1 V to 0.1 V. Here, the linear response clarifies that the kink is not induced by the graphene-Au based Schottky effect [19]. By using the expression *σ = dI_sd_/dV_sd_*, in Supplementary Figure 6a (red curve) we plot also the conductivity (*σ*) of our device.

The Fermi level of graphene can be written as *|E_F_|≈ ℏ|v_F_|(πN)^−1/2^*, where *N* is the carrier density, ℏ is the reduced Planck’s constant and *v_F_* is the Fermi velocity. Thus, one can calculate the *E_F_* of a device as a function of *V_sd_*, which changes the carrier concentration *N* based on the above discussion (reply to point 1). In our device, we use *N = ε_0_ε_g_|V*-*V_Dirac_|/ed* to calculate the carrier density. Here *ε_0_* and *ε_g_* are the permittivity in vacuum and in graphene respectively, *V_Dirac_* represents the source-drain voltage needed to keep the graphene at the Dirac point (i.e, the kink in the I-V curve), *e* is the electron charge and *d* is the graphene thickness [20]. Therefore, the mobility of graphene can be determined using the Drude model *σ = eNμ*, where *σ* is the conductivity, *μ* is the mobility, *e* is the elementary charge and *N* is the carrier density. The calculated mobility is shown in Supplementary Figure 6b and we obtain ≈ 1800 cm^2^V^−1^ s^−1^, a typical value for CVD samples [21].


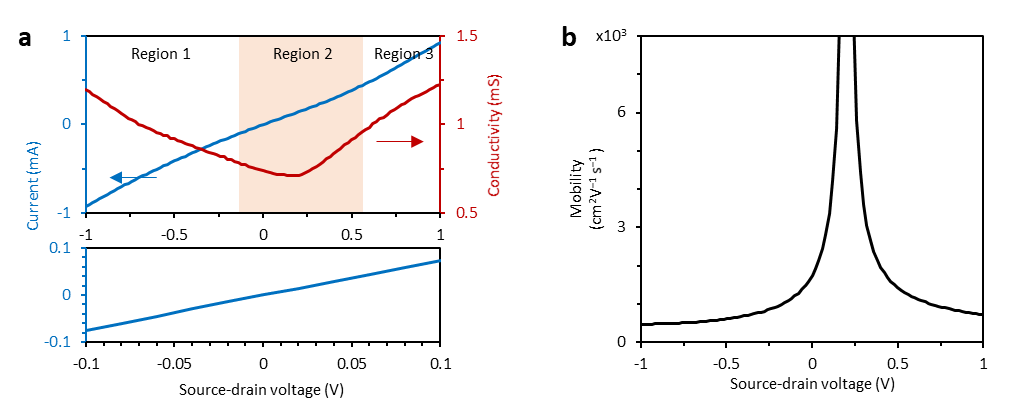


Supplementary Figure 6 | Electrical measurements and discussion. a, I-V curve and conductivity of the GDF device, the bottom panel is a zoom of the source-drain current. b, Calculated mobility of the GDF.

Besides the I-V measurement, we characterize the |*E*_F_| of our graphene device for different values of the *V****_sd_*** voltage by means of in-situ Raman spectroscopy (Renishaw InVia: 514 nm laser excitation, on-sample power <1 mW, integration time 1 s). Supplementary Figure 7a plots three typical Raman spectra of our GDF device (no external voltage, measured at 3 different locations), the ratio of <1/2 between the G and 2D peaks (in Lorentzain shape) reveal the single layer nature of our graphene sample [22]. The half-width of the G peak at ≈ 1586 cm^-^*^1^* is ≈ 26 cm^-^*^1^, the* 2D peak is at ≈ 2681 cm^-^*^1^* with FWHM ≈ 40 cm^-^*^1^*. The wet-transfer technique based on-fiber deposition enables phonon coupling and induces defects at some positions. For example , we observe a clear D peak at 1340 cm^-^*^1^*, a small D+D’’ peak at 2462 cm^-^*^1^*, and a minor D+D’ peak at 2961 cm^-^*^1^*, at the point shown in the top panel of Supplementary Figure 7a. These asymmetric phonon scattering peaks also contribute to the χ^(2)^ and to the plasmon-phonon coupling. But generally, D peak is smaller than 1.2x10^3^ a.u., suggesting the graphene quality is acceptable [23].

Besides *I-V* measurement, we characterize the |*E_F_*| of our graphene device for different values of the *V_D_* voltage by means of *in-situ* Raman spectroscopy (Renishaw InVia: 514 nm laser excitation, on-sample power <1 mW, integration time 1 s). Supplementary Figure 7a plots three typical Raman spectra of our GDF device (no external voltage, measured at 3 varied locations), the <1/2 G and 2D peaks reveal the single layer graphene nature. Typically, the half-width of the G peak at ≈ 1586 cm^-1^ is ≈ 26 cm^-1^, and the 2D peak is at ≈ 2681 cm^-1^, with FWHM ≈ 40 cm^-1^. The G/2D intensity ratio is ≈ 0.5. The wet-transferring technique based on-fiber deposition enables phonon couplings and induces defects at some positions. For example , we observe a clear D peak (178 a.u. intensity) at 1340 cm^-1^, a small D+D’’ peak at 2462 cm^-1^, and a minor D+D’ peak at 2961 cm^-1^, at the point shown in the top panel of Supplementary Figure 7a. These asymmetric phonon scattering peaks also contribute to the χ^(2)^ and the plasmon-phonon coupling. But generally, the graphene quality is acceptable.

Supplementary Figure 7 | In-situ Raman spectroscopy measurements. a, Raman spectra of the GDF. The positions of the D, G, D’, D+D’, D+D’’ and 2D peaks are marked on the graphs. b, Measured Raman spectra at different V_sd_ for the G peak (top) and the 2D peak (bottom). Here the V_sd_ is tuned from 0 V to 0.5 V and 1 V, corresponding to |E_F_| tuning from < 0.1 eV to ≈ 0.4 eV.

To characterize the V_D_ based Fermi level tuning, we plot the in-situ modification of the G peak and 2D peak obtained from measurement and determined by the density functional theory [20]. The shift of the Fermi level induced by V_D_ enables a spectral shift for both the G peak and the 2D peak, depending on the carrier density. When the V_D_ increases from 0.16 V to 1 V, as mentioned in the main text, we estimate the |E_F_| of the graphene on fiber to be in the range 0 eV to 0.4 eV due to external electrons injection. To verify this, Supplementary Figure 7b and Supplementary Figure 7c plot the G peak and the 2D peak for different values of V_D_. When V_D_ = 0 V, 0.5 V, and 1 V, the G peak shifts from 1588 cm^-1^ to 1594 cm^-1^, while the 2D peak shifts from 2681 cm^-1^ to 2675 cm^-1^. In this process, the G peak intensity increases, but the 2D peak intensity decreases, and thus the I_G_/I_2D_ ratio changes from 0.7 to 1.

**Supplementary Note 3. Experimental set-up and extended measurements.**

Supplementary Figure 8 shows our f-2f frequency comb (probe) covering the NIR~MIR band. Typically, the DFG signal that satisfies the phase-matching condition is found by scanning the pump wavelength, although an ultra-broadband (e.g., from C band to MIR band) tunable laser is hard to obtain. On the other, a supercontinuum laser comb perfectly meets the requirement of DFG. In addition, because the DFG signals are weak, previous studies often used lock-in amplification and temporal integration [10,24] for the detection. In this work, two synchronized combs with the same repetition are overlapped in time and offer a sufficiently high peak power for on-line DFG detection.

First, a stabilized mode locked laser with central wavelength 1560 nm is used as the pump. The same laser is super-continuum broadened to an f-2f comb inside a highly nonlinear fiber (HNLF). We show the home-made comb device in Supplementary Figure 8a: this could be realized in a compact and turn-key device. The FROG maps of the laser comb before and after supercontinuum broadening are shown in Supplementary Figure 8b. The retrieved pulse width for the pump and probe combs are ≈ 430 fs and ≈ 110 fs respectively. Here Supplementary Figure 8c plots the electrical self-beating notes of the laser comb source: the repetition is 38 MHz, and the SNR of the first beating-line is higher than 70 dB. Supplementary Figure 8d plots the typical relative intensity noise (RIN) of our frequency comb source: < -100 dBc/Hz at 1kHz and < -140 dBc/Hz at 500 kHz. In the range 0 ~ 1 MHz, the total RIN is < 115 dB. This shoes that the power of the pump and probe pulses is highly stable, ensuring high accuracy for the plasmonic detection via measurement of the ΔI_DFG_.


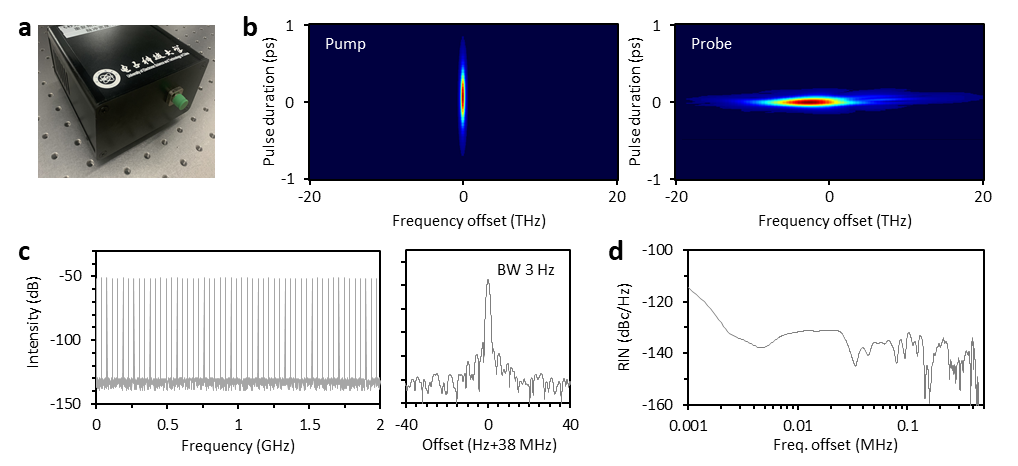


Supplementary Figure 8 | Characterization of the fiber laser frequency combs. a, Device picture and FROG maps before and after supercontinuum generation. b, Measured self-beat-note of the comb source, in the range 0 ~ 1 GHz. Here over 200 beating lines are demonstrated. The SNR of each is > 70 dB, demonstrating the high stability of the light source. c, Measured RIN of the comb source (free running).


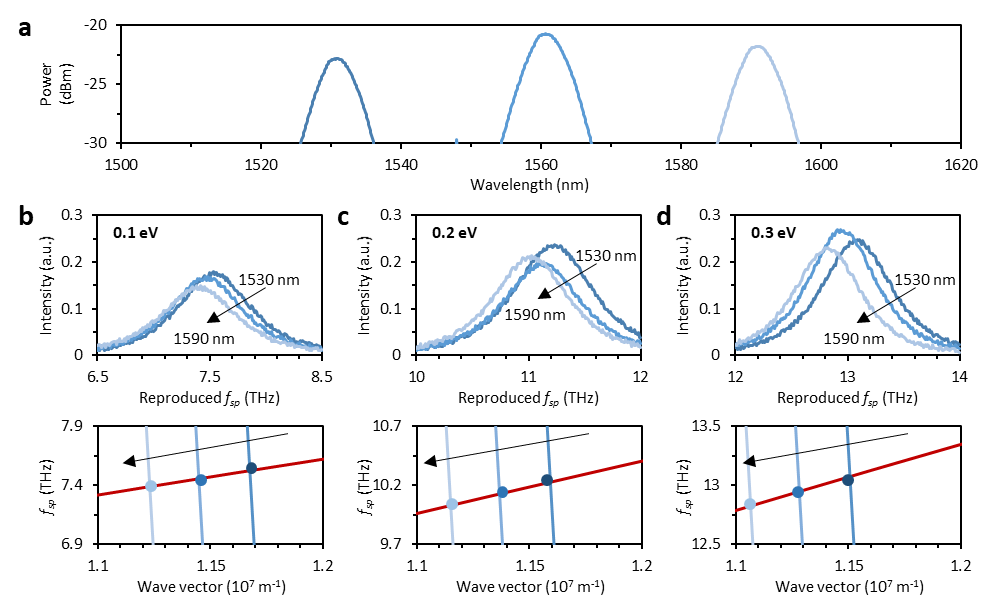


Supplementary Figure 9 | Tuning of the pump wavelength and measurement of the dispersion change. a, Tuning of the pump laser combs, with central wavelengths 1530 nm, 1560 nm and 1590 nm. b to d, Measured plasmonic DFG. Panels from left to right are for *E*_F_ = 0.1 eV, 0.2 eV and 0.3 eV. In each sub-figure, the top panel shows the reproduced plasmonic oscillations, while the bottom panel shows the phase matching space.

The phase matching for DFG of graphene plasmons has been already discussed in the past [10,24,25]. In order to further verify the phase matching conditions in our experiment using two pulsed light sources, we tune the central wavelength of the pump laser (*λ_p_*) at 1530 nm, 1560 nm and 1590 nm (Supplementary Figure 9a) and measure the plasmon DFG for different values of the graphene *E*_F_. Supplementary Figure 9b to Supplementary Figure 9d show the plasmonic oscillation peak of the lowest dispersion branch, when changing the *λ_p_* from 1530 nm to 1590 nm and for *E*_F_ = 0.1 eV, 0.2 eV and 0.3 eV. It is clear that the *f_sp_* shifts down when tuning the *E_F_*. In particular, for increasing *λ_p_* from 1530 nm to 1590 nm, when *E*_F_ = 0.1 eV, the *f_sp_* decreases from 7.54 THz to 7.4 THz, when *E*_F_ = 0.2 eV, the *f_sp_* decreases from 11.22 THz to 11.02 THz and when *E*_F_ = 0.3 eV, the *f_sp_* decreases from 13.08 THz to 12.82 THz. In this figure, we also show the phase matching condition (bottom panels). Here the red curve plots the graphene dispersion in the GDF and the blue curves plot the calculated pump-probe dispersions. The dots show the measured phase-matched points, in good agreement with our model.

Supplementary Figure 10a and 10b show our experimental set-up for the DFG based plasmonic generation. The pump comb is launched into the GDF device from left to right, its peak power can be amplified up to 2kW, corresponding to a peak power density > 4 GW/cm^2^ inside the fiber core. By further spanning this comb via EDFA and highly nonlinear fiber with dispersion compensation, we obtain the probe comb, which covers the wavelength region 1100 nm - 2200 nm, with outstanding spectral flatness in the region 1600 nm - 2000 nm. The probe comb is launched into the GDF device from right to left. The polarization of the counter launched light is fixed to TM by using a pair of fiber polarization controllers. The two light beams are coupled in by using two 1:9 couplers. The GDF is fixed in a temperature controlled chamber to reduce ambient noise. A four-probe electrical stage integrated inside the chamber is used to apply the driving voltage. We tune the temporal delay between the pump and probe pulses by using a high precision motorized delay line (Thorlabs OD 220/M).

When the two pulses overlap in time and the phase-matching condition is satisfied, DFG will occur and we can detect the ΔI_DFG_ on the probe comb spectrum, while a plasmon is generated in the THz band. The plasmon frequency f_sp_ is equal to the difference between the pump and probe frequencies (energy conservation). To further verify the DFG process when the two pulses overlap in time, we verify that ΔI_DFG_ is modulated by the pump comb. As shown in Supplementary Figure 10a, we can modulate at 500 kHz the pump pulse via an AOM and we observe the same modulation on the ΔI_DFG_, as shown in Supplementary Figure 10c. Such modulation only appears at the specific frequency satisfying the phase matching condition.


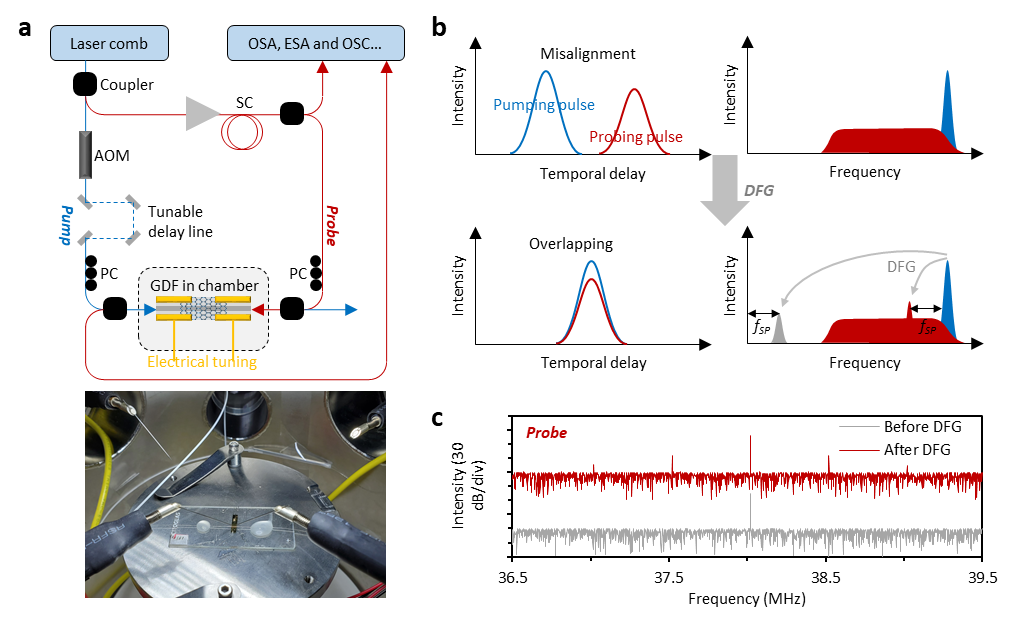


Supplementary Figure 10 | Experimental set-up and DFG. a, Sketch of the counter-pumped set-up. AOM: amplitude optical modulator, SC: super-continuum, PC: polarization controller, OSA: optical spectrum analyzer, ESA: electrical spectrum analyzer, OSC: oscilloscope. b, Schematic of the experimental operation, when the two pulses overlap in time, the DFG occurs and we detect the ΔI_DFG_ at specific frequencies. c, Measured beat note spectrum of the probe comb. Besides the repetition frequency lines (n×38 MHz), a 500 kHz modulation in induces via modulation of the pump comb, as expected for the DFG process.

We also verify that the plasmons are generated in graphene rather than at the gold electrodes. The DFG process can’t occur at the gold contacts for two main reasons. First, the gold electrodes in our device are not in contact with the fiber’s core, hence there is no optical overlap that allows for DFG. Second, the plasmonic dispersion of gold is very different from that of graphene. For instance, at *k* = 10^7^ m^-1^, the typical plasmon frequency of gold is > 300 THz, which is orders of magnitude higher than the *f_sp_* of graphene [26]. Supplementary Figure 11a shows the *∆I_DFG_* in a device without graphene (only gold contacts): here there is no evidence of plasmon generation.

Another important aspect to consider is the thermal effect. The thermal effects induced by the source-drain voltage are negligible because the driving voltage is relatively small (±1V). Considering the Joule heating *P = IV*, the typical mass of our GDF device (3.3 μg) and the typical heat capacity of the fiber (800 J kg^-1^ K^-1^) [14], we can estimate the temperature increment as a function of the source-drain voltage. This is now plotted in the new Supplementary Figure 11b. Even for *V_sd_* = 1V (current in the channel 0.9 mA), the temperature increment is < 260 mK. To show this, we placed the device in a controlled environment by placing a TEC in a vacuum chamber. Here, we can monitor the thermal image (using a thermal camera, FLIR) of the device, as we change *V****_sd_***. As expected, we observe that the temperature in the channel does not increase remarkably by tuning the source-drain voltage (see Supplementary Figure 11c).


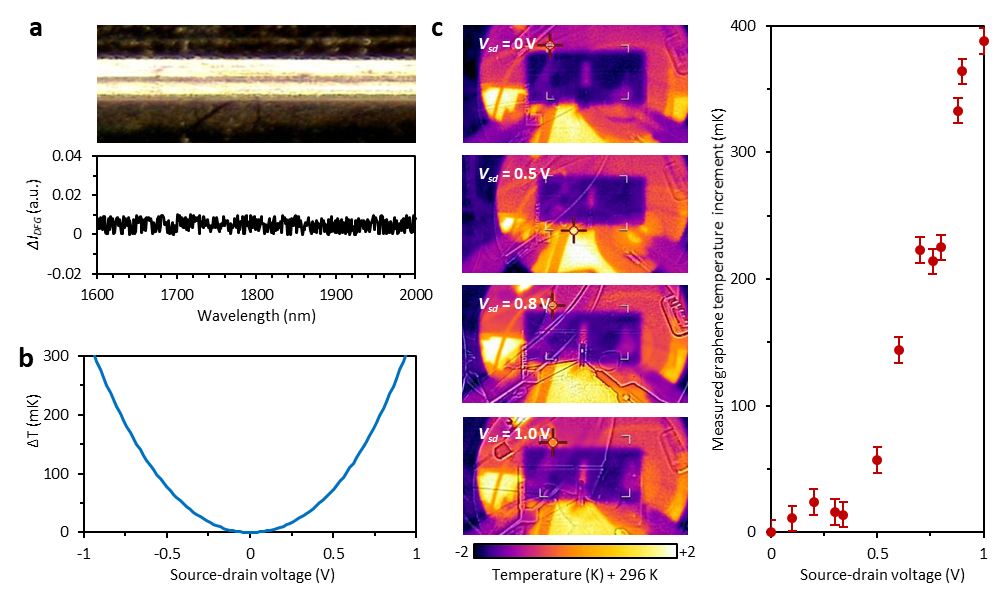


Supplementary Figure 11 | Role of gold contacts and thermal effects. a, DFG in an Au covered D shaped fiber. Top: Optical microscope image of the device, Bottom: ΔI_DFG_, measured in the optical band 1600 nm to 2000 nm. b, Calculated Joule heating effect in the device. c, Measured thermal images and temperature curve. The error bar represents the measurement uncertainty of the FLIR (±10 mK).

As mentioned in the main text, the three logic gates (AND, OR, NOR) are obtained by selective filtering of the DFG signal. Thanks to our all-fiber system, we can use three band-pass filters (BPF) based on fiber-Bragg gratings to select the three ΔI_DFG_ at different frequencies. Supplementary Figure 12a shows the reflection spectra of the three BPFs at the output port of the probe. BPF1 is used as ‘NOR’ gate, with central wavelength 1624 nm and bandwidth 2 nm, BPF2 is used as ‘OR’ gate, with central wavelength 1815 nm and bandwidth 1.8 nm, BPF3 is used as ‘AND’ gate, with central wavelength 1930 nm and bandwidth 2.4 nm. The central wavelength of the filters can be slightly tuned via a piezo (≈ ± 5 nm) to optimize the detection of the ΔI_DFG_ signal. Supplementary Figure 12b illustrates the reflected intensity uncertainty (<5%) of the three BPFs for a 5 minutes measurement while Supplementary Figure 12c shows the extinction ratio of the three BPFs.

Supplementary Figure 12 | Characterization of the bandpass filters. a, Reflection spectra of the 3 BPFs, which are used as outputs for the logic gates NOR, OR, AND. Here the pink, yellow and dark blue shadows mark the selected ΔI_DFG_ regions when the GDF operates in the NOR, OR, AND states respectively. b, Intensity uncertainty of the three BPFs. c, Measured extinction ratio of the three BPFs, the extinction ratio of each BPF is much larger than its uncertainty.

**Supplementary references**

1. Mikhailov, S. A. & Ziegler, K. New electromagnetic mode in graphene. *Phys. Rev. Lett.* **99**, 016803 (2007).

2. Vakil, A. & Engheta, N. Transformation optics using graphene. *Science* **332**, 1291–1294 (2011).

3. Bonaccorso, F., Sun, Z., Hasan, T. & Ferrari, A. C. Graphene photonics and optoelectronics. *Nat. Photonics* **4**, 611–622 (2010).

4. Yao, B. *et al.* Gate-tunable frequency combs in graphene–nitride microresonators. *Nature* **558**, 410–414 (2018).

5. Grigorenko, A. N., Polini, M. & Novoselov, K. S. Graphene plasmonics. *Nat. Photonics* **6**, 749–758 (2012).

6. Basov, D. N., Fogler, M. M. & Garcia de Abajo, F. J. Polaritons in van der Waals materials. *Science* **354**, aag1992–aag1992 (2016).

7. Hwang, E. H., Sensarma, R. & Das Sarma, S. Plasmon-phonon coupling in graphene. *Phys. Rev. B* **82**, 195406 (2010).

8. Low, T. & Avouris, P. Graphene Plasmonics for Terahertz to Mid-Infrared Applications. *ACS Nano* **8**, 1086–1101 (2014).

9. Kotov, V. N., Uchoa, B., Pereira, V. M., Guinea, F. & Castro Neto, A. H. Electron-Electron Interactions in Graphene: Current Status and Perspectives. *Rev. Mod. Phys.* **84**, 1067–1125 (2012).

10. Yao, B. *et al.* Broadband gate-tunable terahertz plasmons in graphene heterostructures. *Nat. Photonics* **12**, 22–28 (2018).

11. Yao, X., Tokman, M. & Belyanin, A. Efficient Nonlinear Generation of THz Plasmons in Graphene and Topological Insulators. *Phys. Rev. Lett.* **112**, 055501 (2014).

12. Ni, G. X. *et al.* Fundamental limits to graphene plasmonics. *Nature* **557**, 530–533 (2018).

13. An, N. *et al.* Electrically Tunable Four-Wave-Mixing in Graphene Heterogeneous Fiber for Individual Gas Molecule Detection. *Nano Lett.* **20**, 6473–6480 (2020).

14. An, N. *et al.* Electrically Tunable Four-Wave-Mixing in Graphene Heterogeneous Fiber for Individual Gas Molecule Detection. *Nano Lett.* **20**, 6473–6480 (2020).

15. Liu, M. *et al.* A graphene-based broadband optical modulator. *Nature* **474**, 64–67 (2011).

16. Schwierz, F. Graphene transistors. *Nat. Nanotechnol.* **5**, 487–496 (2010).

17. Meric, I. *et al.* Current saturation in zero-bandgap, top-gated graphene field-effect transistors. *Nat. Nanotechnol.* **3**, 654–659 (2008).

18. Di Bartolomeo, A. Graphene Schottky diodes: An experimental review of the rectifying graphene/semiconductor heterojunction. *Phys. Rep.* **606**, 1–58 (2016).

19. Liu, Y. *et al.* Approaching the Schottky–Mott limit in van der Waals metal–semiconductor junctions. *Nature* **557**, 696–700 (2018).

20. Das, A. *et al.* Monitoring dopants by Raman scattering in an electrochemically top-gated graphene transistor. *Nat. Nanotechnol.* **3**, 210–215 (2008).

21. Banszerus, L. *et al.* Ultrahigh-mobility graphene devices from chemical vapor deposition on reusable copper. *Sci. Adv.* **1**, e1500222 (2015).

22. Ferrari, A. C. *et al.* Raman Spectrum of Graphene and Graphene Layers. *Phys. Rev. Lett.* **97**, 187401 (2006).

23. Cançado, L. G. *et al.* Quantifying Defects in Graphene via Raman Spectroscopy at Different Excitation Energies. *Nano Lett.* **11**, 3190–3196 (2011).

24. Constant, T. J., Hornett, S. M., Chang, D. E. & Hendry, E. All-optical generation of surface plasmons in graphene. *Nat. Phys.* **12**, 124–127 (2016).

25. Cox, J. D. & García de Abajo, F. J. Nonlinear Graphene Nanoplasmonics. *Acc. Chem. Res.* **52**, 2536–2547 (2019).

26. Lyvers, D. P., Moon, J.-M., Kildishev, A. V., Shalaev, V. M. & Wei, A. Gold Nanorod Arrays as Plasmonic Cavity Resonators. *ACS Nano* **2**, 2569–2576 (2008).
