# Supplementary material for: Nonlinear co-generation of graphene plasmons for optoelectronic logic operations
Source: Nat Commun. 2022 Jun 6;13:3138. doi: 10.1038/s41467-022-30901-8 (PMC9170737; doi:10.1038/s41467-022-30901-8)
Supplement: Supplementary file 3 — Description of Additional Supplementary Files [file 41467_2022_30901_MOESM3_ESM.docx]

**Description of Additional Supplementary Files**

**Legend of Supplementary Movie 1:**

Monitoring the DFG based signal enhancement in the auto-correlation measurement
